# Acute intermittent hypoxia and rehabilitative training following cervical spinal injury alters neuronal hypoxia- and plasticity-associated protein expression

**DOI:** 10.1371/journal.pone.0197486

**Published:** 2018-05-18

**Authors:** Atiq Hassan, Breanna M. Arnold, Sally Caine, Behzad M. Toosi, Valerie M. K. Verge, Gillian D. Muir

**Affiliations:** 1 Department of Biomedical Sciences, WCVM, University of Saskatchewan, Saskatoon, Saskatchewan, Canada; 2 Cameco MS Neuroscience Research Center, University of Saskatchewan, Saskatoon, Saskatchewan, Canada; 3 Department of Anatomy and Cell Biology, College of Medicine, University of Saskatchewan, S askatoon, Saskatchewan, Canada; Imperial College London, UNITED KINGDOM

## Abstract

One of the most promising approaches to improve recovery after spinal cord injury (SCI) is the augmentation of spontaneously occurring plasticity in uninjured neural pathways. Acute intermittent hypoxia (AIH, brief exposures to reduced O_2_ levels alternating with normal O_2_ levels) initiates plasticity in respiratory systems and has been shown to improve recovery in respiratory and non-respiratory spinal systems after SCI in experimental animals and humans. Although the mechanism by which AIH elicits its effects after SCI are not well understood, AIH is known to alter protein expression in spinal neurons in uninjured animals. Here, we examine hypoxia- and plasticity-related protein expression using immunofluorescence in spinal neurons in SCI rats that were treated with AIH combined with motor training, a protocol which has been demonstrated to improve recovery of forelimb function in this lesion model. Specifically, we assessed protein expression in spinal neurons from animals with incomplete cervical SCI which were exposed to AIH treatment + motor training either for 1 or 7 days. AIH treatment consisted of 10 episodes of AIH: (5 min 11% O_2_: 5 min 21% O_2_) for 7 days beginning at 4 weeks post-SCI. Both 1 or 7 days of AIH treatment + motor training resulted in significantly increased expression of the transcription factor hypoxia-inducible factor-1α (HIF-1α) relative to normoxia-treated controls, in neurons both proximal (cervical) and remote (lumbar) to the SCI. All other markers examined were significantly elevated in the 7 day AIH + motor training group only, at both cervical and lumbar levels. These markers included vascular endothelial growth factor (VEGF), brain-derived neurotrophic factor (BDNF), and phosphorylated and nonphosphorylated forms of the BDNF receptor tropomyosin-related kinase B (TrkB). In summary, AIH induces plasticity at the cellular level after SCI by altering the expression of major plasticity- and hypoxia-related proteins at spinal regions proximal and remote to the SCI. These changes occur under the same AIH protocol which resulted in recovery of limb function in this animal model. Thus AIH, which induces plasticity in spinal circuitry, could also be an effective therapy to restore motor function after nervous system injury.

## Introduction

Spinal cord injury (SCI) damages axonal pathways, interrupting synaptic transmission between the brain and spinal cord and subsequently altering motor, sensory and autonomic functions below the level of injury. Most SCIs are incomplete, and the sparing of undamaged pathways contributes to spontaneous recovery of some limb and respiratory function following SCI. Nevertheless, this recovery is frequently inadequate to restore normal function. A variety of approaches have been used to enhance functional recovery in animal models of SCI, including methods to facilitate plasticity in uninjured neural pathways throughout the brain and spinal cord [1].

Acute intermittent hypoxia (AIH) is one approach known to induce plasticity in multiple physiological systems [2]. Intermittent hypoxia involves exposure of persons or animals to short periods of low oxygen levels. Beneficial effects of acute or low-dose exposure to intermittent hypoxia has been most thoroughly studied in the respiratory system, where brief (<5 min) exposures to reduced oxygen levels (~10.5% inspired O_2_), alternating with exposure to normal levels (20% O_2_), results in a sustained increase in the output of phrenic motoneurons for several hours after the stimulus has ended [2, 3]. This increase is known as long-term facilitation (LTF) and is potentially a manifestation of a compensatory mechanism which stabilizes respiratory motor output following hypoxia exposure [1, 3–5].

The mechanism of action of AIH is complex, but it has been well established that multiple convergent intracellular pathways are responsible for AIH-induced LTF within spinal neurons (for review, see [1]). These pathways are mainly described as “Q” and “S” pathways, based on the type of G protein (Gq- and Gs) with which the response-initiating metabotropic receptors are coupled. Moderate AIH elicits LTF through the Q pathway, which requires spinal serotonin type 2 receptor (5-HT2) activation, protein kinase (PK) C-mediated increase in brain-derived neurotrophic factor (BDNF) synthesis and activation of its high-affinity receptor, tropomyosin-related kinase B (TrkB), followed by ERK/MAP kinase activity [1]. However, the S pathway signaling is mostly activated in response to more severe AIH conditions and is characterized by spinal adenosine 2A and 5-HT7 receptor activation, formation of cyclic adenosine monophosphate (cAMP) and synthesis of an immature TrkB isoform (BDNF-independent) and downstream signaling via the phosphatidylinositol 3-kinase/protein kinase B (PI3K/Akt) pathway [3, 6]. As an alternate pathway, it has been shown that vascular endothelial growth factor (VEGF) is also able to elicit LTF by acting through its high affinity VEGF type 2 receptor (VEGFR2), and by the activation of both ERK/MAP kinase and Akt signaling (for review, see [1]). Both VEGF and VEGFR2 are expressed in motor neurons and their expression is regulated by hypoxia-inducible factor-1α (HIF-1α), a transcriptional activator which mediates a range of systemic and cellular responses to hypoxia [7].

While these pathways have been shown to induce LTF in anesthetized, ventilated rodent preparations, it is not yet clear whether the same mechanisms are responsible for the positive behavioural effects of AIH administered in animals or humans with SCI. AIH protocols administered in animal SCI models and in persons with SCI have been shown to have beneficial effects for both respiratory and non-respiratory motor systems. For example, repetitive exposure to AIH (daily for 7 days) improves respiratory tidal volume in rats with cervical hemisection [8]. Indeed, we have shown that the same 7 day AIH exposure in cervical spinal-injured rats will also improve performance on a horizontal ladder task. Rats with a unilateral transection of the dorsolateral funiculus at the second cervical spinal segment (C2) are able to cross a horizontal ladder but will consistently make foot-slip errors with the forepaw ipsilateral to the injury [8, 9]. When these same rats are subject to 7 days of AIH treatment starting 4 weeks post-injury, AIH-treated animals make fewer foot-slip errors compared to control-treated animals [8]. We have more recently shown that this AIH-induced improvement in ladder performance requires that animals receive motor rehabilitative training concurrently with AIH treatment [9]. Remarkably, AIH exposure also improves motor function in persons with SCI. In people with incomplete chronic spinal cord injuries, a single AIH exposure increases ankle strength; furthermore, daily exposure to AIH for 5 days enhances walking speed and endurance when combined with walking training [10, 11].

AIH is therefore a promising therapy which has the potential to improve motor recovery after SCI. Nevertheless, the mechanisms by which AIH exerts functional effects in spinal-injured animals and humans are as yet unknown. As a first step, it was previously demonstrated that several key proteins involved in the intracellular pathways described above, i.e. those pathways underlying AIH-induced respiratory plasticity in the form of LTF, are altered by AIH exposure [12, 13]. In awake uninjured rats, AIH exposure 3 times per week for 10 weeks resulted in increases in spinal serotonin (type 2A) receptor, BDNF and its high affinity receptor TrkB, vascular endothelial growth factor A (VEGF-A) and its high-affinity receptor (VEGFR-2), and HIF-1α [12, 13]. Interestingly, AIH altered protein expression in neurons situated bilaterally and in multiple locations along the spinal cord. The effects of AIH are not restricted to the spinal cord–in a separate study using mice, AIH increased HIF-1α and VEGF levels in sensory neurons in dorsal root ganglia, and promoted axonal regeneration in a HIF-1α -dependent manner [14].

The purpose of this experiment was to investigate whether our AIH protocol (a combination of 7 days AIH + rehabilitative training, which we previously showed to improve forelimb function in cervically injured rats [8, 9]) is also associated with altered plasticity- and hypoxia- related protein expression in spinal neurons. Here, we used a sham treatment-controlled, randomized study to examine the effect of AIH exposure on the response of spinal neurons in multiple locations along the spinal cord. Our results show a rapid significant early increase in expression of the hypoxia-associated transcription factor in AIH-treated rats relative to normoxia controls in spinal neurons both proximal and remote to the incomplete cervical SCI. This increase was still evident following 7 days of therapy, with increased plasticity-related protein expression only observed after 7 days of treatment. These findings revealed an early impact of AIH in regulation of hypoxia-related transcription factor expression that was then followed by a more protracted regulation of plasticity-associated gene expression.

## Materials and methods

### Animals and experimental design

Twenty-four male Lewis rats (225-250g, Charles River Laboratories, Quebec) were housed 3 rats/cage upon arrival to the facility and allowed to acclimate to the colony room for 5 days prior to handling. Temperature of the room was maintained at 20°C and lights were on an automated cycle of 12hL: 12hD at the Animal Care Facility, Western College of Veterinary Medicine, University of Saskatchewan. Cages (51 x 28cm) contained wood chip bedding, PVC tubes for hiding and sleeping in, and wood blocks for chewing. Rats were fed rodent chow *ad libitum* until an approximate weight of 320g was reached, at which time they were restricted to 4 pellets/rat/day for duration of the study. Rats had *ad libitum* access to water throughout the study. Prior to initiation of behavioural training, rats were handled gently 10 min/day for approximately 3 days or until deemed comfortable with the handler. Individual animals were handled by the same person for all procedures. All animal procedures were approved by the University of Saskatchewan Committee on Animal Care and Supply and carried out in accordance with standards set out by the Canadian Council on Animal Care.

**Fig 1:**
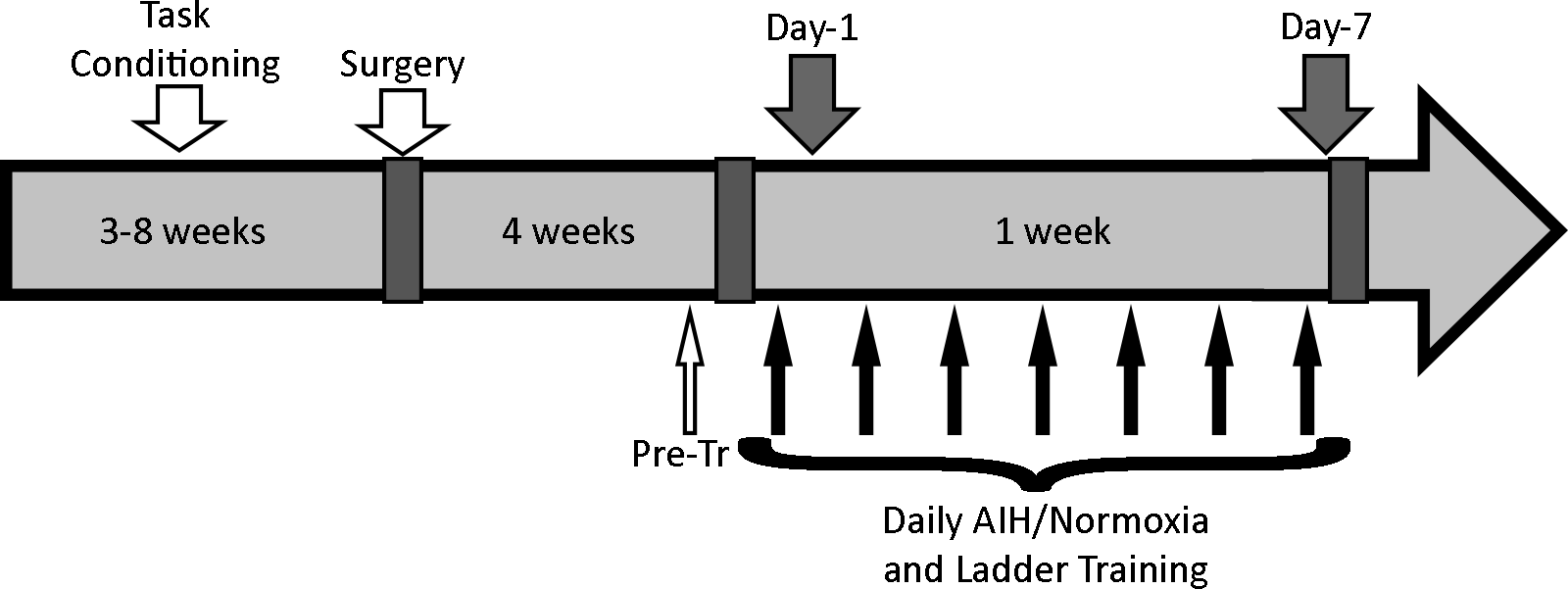
Experimental design. All animals were initially conditioned on the ladder task for 3–8 weeks, underwent spinal surgery and were assessed on the ladder task 4 weeks after surgery but before treatment (**Pre-tr**). Animals then received AIH or normoxia treatment plus ladder training for one day (**Day 1**) or every day for 7 days **(Day 7**). Spinal cord tissue was taken from half of the animals (n = 6/treatment group), which were euthanized and perfused after 1 day of treatment and training (**Day 1**), and from the remaining animals (n = 6/treatment group) after 7 days of treatment and training (**Day 7**).

The experimental timeline is shown in Fig 1. All rats were initially conditioned on the ladder task prior to surgery for unilateral transection of the dorsolateral funiculus at the C2 level. The location and size of spinal transection injury was chosen because it produces a consistent foot-slip deficit of the ipsilateral forepaw during the horizontal ladder task [8, 9, 15]. Four weeks after surgery, performance on the ladder task was evaluated and animals were grouped based on their ladder task deficit such that the deficits were matched between the groups. The groups were randomly assigned to receive either AIH or control (normoxia) treatment (n = 12 per treatment group). This ensured equivalence of lesion severity between AIH and normoxia treatment groups. Within each treatment group, animals were randomly assigned to receive either 1 day or 7 days of treatment plus ladder training. The experimental groups were therefore 1 day normoxia; 7 day normoxia; 1 day AIH; or 7 day AIH (n = 6 per experimental group). During the treatment week, rats underwent ladder training 1 hour after each treatment.

### Ladder-walking task

Rats were trained to cross a horizontal runway, consisting of a ladder apparatus 120 cm in length, with 20 cm opaque plexiglass platforms at either end allowing the rats to turn around. The central ladder portion (80 cm in length) consisted of 2 mm diameter wire rungs spaced 2 cm apart. The ladder was positioned above a 45° angled mirror so both lateral and ventral aspects of the rat movements were visible in the digital video camera (EOS Rebel, T2i EOS 550D Canon) positioned perpendicular to the runway.

Ladder conditioning and training took place at the same time each day for each group of animals. Animals were initially conditioned to walk on the runway for 3–8 weeks, after which time they would repeatedly cross the ladder consistently and quickly with very few long pauses or hesitations. Training on the ladder-task was carried out at the following time points: pre-surgery, pre-treatment, and each of the days of treatment. All training sessions involved 12 complete crossings.

### Surgery

Animals were placed under isoflurane anaesthesia and administered an antibiotic (trimethoprim and sulfadoxine (TMS), Trivetrin, Schering Canada Inc., QC, Canada, 30mg/kg sub-cutaneous (SC)) and pre-emptive analgesia (buprenorphine, Buprenex; Reckitt Benckiser Pharmaceuticals Inc., Richmond, VA, USA, 0.05mg/kg SC). The surgical site over the dorsum of the neck was prepared by removing fur with clippers and cleaning the skin with stanhexidine and 70% isopropyl alcohol. Under an operating microscope, the spinal laminae of the 2^nd^ and 3^rd^ cervical vertebrae were exposed using sterile technique, and a laminectomy and durotomy performed to expose the 2^nd^ cervical spinal segment. The dorsolateral funiculus was transected unilaterally on the left side using a modified 25-gauge bevel tipped needle. The muscle was closed with a simple continuous suture, and the skin was closed with a subcuticular suture technique. The entire time spent under anaesthetic was approximately 20–30 min. Following surgery, rats were administered 3 ml warm sterile saline (SC) and housed individually in cages equipped with wood chip bedding, a plastic tube and an extraneous heat source. Post-operative analgesia (buprenorphine 0.05mg/kg) and antibiotic (TMS, 30mg/kg) were administered for 48h post-surgery, and longer if necessary. Rats were monitored several times daily for 5 days post-surgery and assessed for change in weight, presence and severity of porphyrin, hydration, healing of the incision site, mobility, and general behavior. At day 3 post-surgery, rats were rehoused with their original cage mates.

### Intermittent hypoxia treatment

Rats were acclimated to the treatment apparatus by placing them for 30 min into custom-made Plexiglas chambers (1 rat per chamber; 30cm × 17cm × 12cm) under normoxia (21% inspired O_2_), 1 day prior to the first treatment day as previously described [8, 9]. Subsequently, on each day of treatment, the rats were placed into the Plexiglas chamber and then exposed to AIH, consisting of ten 5 min hypoxic episodes (11% inspired O_2_), alternating with 5 min normoxic intervals. Alteration in normoxic and hypoxic conditions were established by automatically switching the incoming air between premixed O_2_ and N_2_ gas (FlO_2_ = 0.11) and medical air (FlO_2_ = 0.21). Animals receiving control treatment (normoxia) were placed in adjacent chambers for the same total duration of time under continuous normoxic conditions (FlO_2_ = 0.21). The oxygen levels in the chambers were continuously monitored using an oxygen analyzer (AX300-1 Portable Oxygen Analyzer; Teledyne Analytical Instruments).

### Tissue preparation

Beginning one hour after the training portion of the treatment on either day 1 or day 7, animals were deeply anaesthetized with isoflurane and perfused trans-cardially with heparinized phosphate-buffered saline followed by cold 4% paraformaldehyde in 0.01M phosphate buffer. The vertebral column was removed and post-fixed overnight in 4% paraformaldehyde. The spinal cord was dissected from the vertebral column and segments C6-7 (containing forelimb motoneurons) and L4-5 (containing hindlimb motoneurons) were postfixed for 1–1.5h in 4% paraformaldehyde followed by cryoprotection in 10% and then 20% sucrose overnight at 4°C, respectively. Subsequently, segments of spinal cord from each of the experimental groups were embedded in the same cryomold covered in OCT compound (Tissue Tek; Miles Laboratories, Elkhart, IN), and frozen in isopentane cooled in a slurry of dry ice and acetone. Thus, each block contained 8 pieces of spinal cord, representing each of the 4 experimental conditions (1 day normoxia; 7 day normoxia; 1 day AIH; 7 day AIH) from each of the spinal cord regions (C6-7 and L4-5). Blocks were then stored at -80°C until sectioning. Tissues were sectioned at 10μm on a Microm cryostat, thaw-mounted onto silanized slides (VWR Superfrost Plus) and stored at -80°C. The presence of representative spinal cord sections from all experimental groups on each slide allowed processing under identical conditions and intergroup analysis within the same slide.

### Immunofluorescence

Slides were brought to room temperature, then washed 3×10 min in 0.01M PBS, pH 7.4. For BNDF and TrkB only, citrate antigen retrieval was performed prior to blocking and incubation with primary antibodies. For this extra step, slides were placed in 0.01M citrate buffer (10% 0.1M sodium citrate buffer in ddH2O, pH 6) at 50°C and then warmed to 90°C over 45min. Slides were then allowed to cool for 20min. For all antibodies, slides were incubated with blocking solution containing undiluted Sea Block buffer (Abcam) for 1hr at room temperature. Primary antibodies were diluted with 10% Sea Block in primary diluent (0.1% Triton X-100 in 0.01M PBS) to the following concentrations: mouse anti- HIF-1α (NB 100–105, Novus Biological) 1:200, rabbit anti-VEGF (sc-152, Santa Cruz Biotech, Inc) 1:200, chicken anti-BDNF (Promega) 1:200, rabbit anti-TrkB (sc-12, Santa Cruz Biotech, Inc) 1:100, rabbit anti phospho-TrkB (rat phospho Y705, Y706, ab111545, Abcam) 1:400. After 24hr incubation with the primary at 4°C in a humidified chamber, slides were washed for 3× 10min in 0.01M PBS. The following secondary antibodies were used: goat-anti-rabbit Alexa Fluor 488, donkey-anti-mouse Alexa Fluor 488 (Jackson Immuno Research Laboratories, Inc) at 1:2000, goat-anti-chicken (F-1005, aves labs, Inc) 1:1000 diluted in 0.01M PBS. After 1hr incubation in the dark at room temperature, slides were washed for 3× 10min in 0.01M PBS, and then coverslipped using ProLong Gold Antifade Reagent (P36931, Molecular Probes, Invitrogen).

For double staining for choline acetyl-transferase (ChAT) and HIF-1α, sections were brought to room temperature, underwent antigen retrieval with citrate buffer as described above and blocked in undiluted Sea Block buffer for 1h. Anti-ChAT (AB144P, Millipore) and anti-HIF-1α (GTX127309, GeneTex) antibodies were diluted at 1:100 and 1:200 respectively in 0.01M PBS with 10% Sea Block and 0.1% Triton X-100 and were applied simultaneously on the sections. Sections were incubated with these antibodies for 24hr at 4°C in a humidified chamber. The slides were subsequently washed 3× 10min in 0.01M PBS before simultaneous application of the secondary antibodies, Alexa Fluor 594 (A-11058, Invitrogen) and Alexa Fluor 488 (A-21206, Invitrogen), diluted 1:500 in 0.01M PBS. Sections were incubated with the secondary antibodies for 1hr in the dark at room temperature. The secondary solution was removed with 3× 10min washes in 0.01M PBS and the nuclei were stained with DAPI (D9542, Sigma-Aldrich) for 10min at room temperature using a 300 nM DAPI staining solution in 0.01M PBS. Finally, the slides were again washed 3 × 10min in 0.01M PBS before coverslipping with a 1:1 glycerol/0.01M PBS solution.

For all markers specificity of the secondary antibodies employed in this study was confirmed in experiments where the primary antibodies were omitted, revealing an absence of nonspecific staining.

### Image analysis / quantification

**Fig 2:**
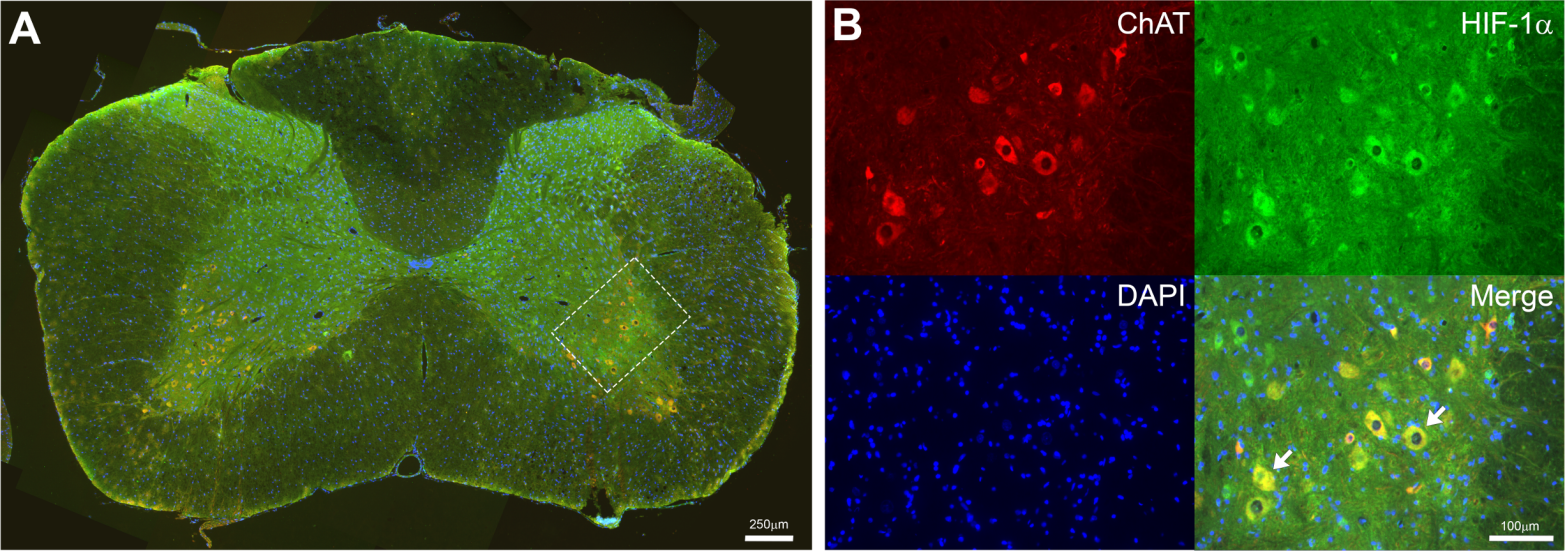
Spinal neurons identified as motoneurons with choline acetyl-transferase (ChAT). Photomicrograph of the spinal cord in C6-7 region (**A**) processed for ChAT, HIF-1α and DAPI immunofluorescence from a 7 day AIH-treated spinal-injured rat. Scale bar = 250 μm. White box shows location of representative images in (**B**) processed for each of ChAT, HIF-1α and DAPI. Merged image shows HIF-1α co-localization with ChAT and DAPI in spinal neurons (e.g. arrows). Scale bar = 100 μm.

Immunofluorescence-processed sections were examined and imaged using a Zeiss Axioskop (20× objective) with all spinal cord ventral cord regions of the different experimental groups imaged under identical conditions. Initially, spinal neurons located on both sides of the cord were analyzed separately and the data were pooled after we determined that there were no differences between the levels of protein expression from side to side. All slides were first qualitatively examined and alterations in immunofluorescence noted. Immunofluorescence signal intensity was quantified for 3 out of 6 animals in each experimental group using Northern Eclipse (Empix Imaging) and FIJI (ImageJ). Briefly, the cell bodies of neurons in ventral grey matter on both sides of the spinal cord were identified by their location and size. Only cells for which the cytoplasmic area visible was equal to or greater than the area of the nucleus were included. Immunofluorescence labeling for ChAT confirmed that these identified neurons were motoneurons (Fig 2). All neurons meeting these criteria were analyzed from 2 separate slides for each grouping (n = 3) of all 8 experimental conditions mounted on the same slide (1 day normoxia C6-7 & L4-5; 7 day normoxia C6-7 & L4-5; 1 day AIH C6-7 & L4-5; 7 day AIH C6-7 & L4-5), representing a total of 60–85 neurons analysed/time point/experimental condition. Quantitative image analysis was performed by an individual blinded to experimental condition of the image being analysed. Neurons were circumscribed manually and the mean gray value for the marker being examined was obtained for the cytoplasm of each neuron. To determine whether neuronal size, and consequently the mean gray value, would be affected by treatment, we compared the mean cell areas (micron^2^) for each experimental condition and found no significant differences between groups. The area of the nuclei was excluded from analysis. Mean gray background values were determined over regions of the neuropil devoid of positive signal and subtracted from the mean gray value for each neuron analyzed in that spinal cord section and then the net mean gray value determined for that grouping of neurons. The mean gray value for each slide was averaged to get the mean gray value for each animal within each experimental group. The mean gray values for each of 3 animals in each of 4 experimental groups (1 day normoxia; 7 day normoxia; 1 day AIH; 7 day AIH) were then averaged to obtain mean gray values for each group at both the cervical and lumbar levels.

Bar graphs were made in Graphpad prism v6.02 (La Jolla, CA) with composite figures compiled using Adobe Photoshop CC software (Adobe System, San Jose, CA).

### Statistical analysis

Statistical analysis was performed with IBM SPSS Statistics v20 for Windows software. Differences between the mean gray values for each experimental group at C6-7 or L4-5 spinal segments for each of BDNF, TrkB, phospho-TrkB, HIF-1α and VEGF were examined by using one-way analysis of variance (ANOVA) and Tukey HSD test was used for post hoc analysis. Differences were considered significant if *p*<0.05.

## Results

### AIH and motor training results in early and sustained increases in HIF-1α protein expression in spinal neurons at cervical and lumbar spinal segments

**Fig 3:**
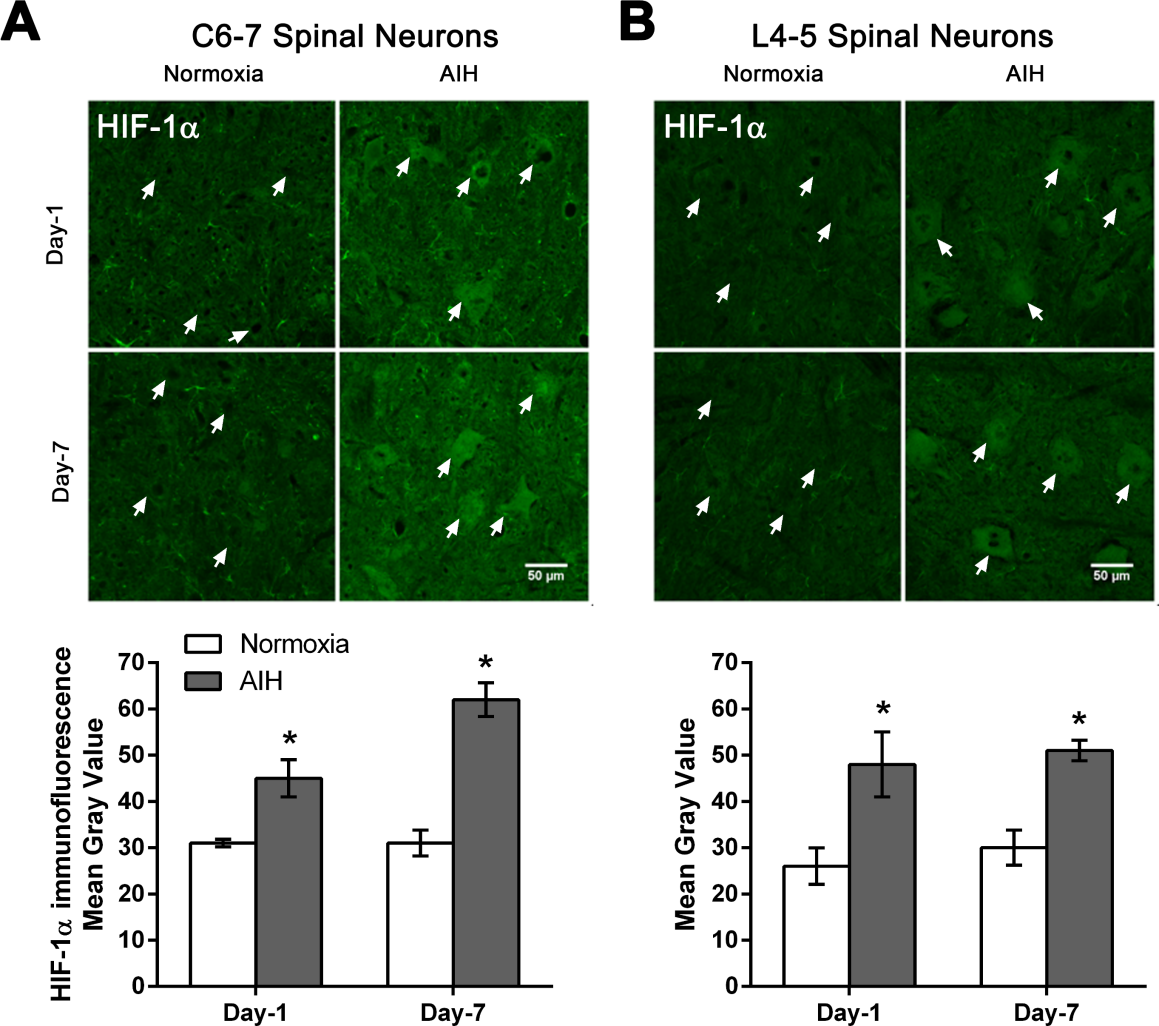
AIH treatment for either 1 or 7 days increased HIF-1α protein levels in multiple spinal segments. Representative photomicrographs of the ventral grey matter in C6-7 (**A**) or L4-5 (**B**) spinal segments sections processed for HIF-1α immunofluorescence from 1 day (Day-1) or 7 day (Day-7) in normoxia-treated versus AIH-treated spinal injured rats. Arrows indicate representative neurons. Scale bar = 50 μm. Histograms summarize the mean immunofluorescence signal intensity detected ± SEM as measured in gray values over individual neurons within the ventral horn from normoxia- and AIH-treated spinal injured rats [n = 3 rats per experimental group analysed; 60–65 neurons analysed/time point/experimental condition at C6-7 (**A**); 80–85 neurons analysed/time point/experimental condition at L4-5 (**B**)]. Asterisks indicate significant differences between experimental groups; * p < 0.05.

HIF-1α is a transcriptional regulator of genes controlling a number of adaptive responses to low oxygen tension in order to maintain oxygen homeostasis in mammalian cells and more recently linked to neurorepair [16, 17]. AIH treatment and motor training for 1 or 7 days increased HIF-1α protein expression in spinal motoneurons at C6-7 and L4-5 segments of spinal cord (Fig 3). Photomicrographs of ventral grey matter region of spinal cord processed for HIF-1α immunofluorescence showed increased immunoreactivity in neurons in AIH-treated + trained SCI rats compared to normoxia-treated + trained SCI rats after 1 day (Day-1). A similar change occurred in response to 7 days (Day-7) of treatment in putative motoneurons (large neurons in ventral horn) of both C6-7 (Fig 3A) and L4-5 (Fig 3B) spinal segments. Quantitative analysis confirmed that AIH treatment plus motor training for either 1 day or 7 days significantly increased HIF-1α protein levels in the neurons of ventral grey matter of C6-7 and L4-5 spinal segments in AIH-treated rats relative to normoxia-treated control rats (p < 0.05) (Fig 3A–3B and S1 Table).

### VEGF protein expression in spinal neurons is increased in response to AIH and motor training for 7 days

**Fig 4:**
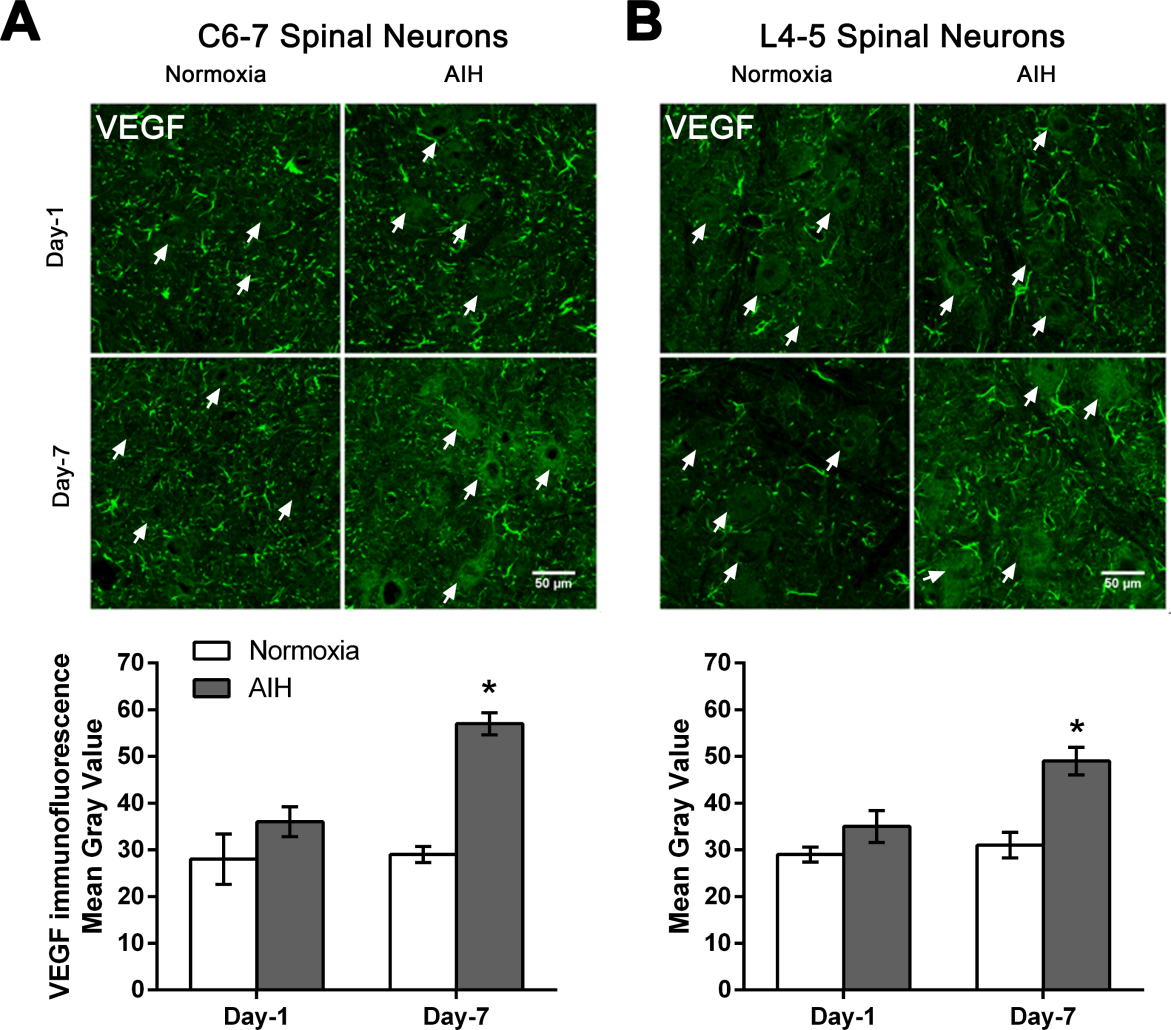
AIH treatment for 7 days increased neuronal VEGF protein levels in multiple spinal segments. Representative photomicrographs of the ventral grey matter in C6-7 (**A**) or L4-5 (**B**) spinal segments sections processed for VEGF immunofluorescence from 1 day (Day-1) or 7 day (Day-7) in normoxia-treated versus AIH-treated spinal injured rats. Arrows indicate representative neurons. Scale bar = 50 μm. Histograms summarize the mean immunofluorescence signal intensity detected ± SEM as measured in gray values over individual neurons within the ventral horn from normoxia- and AIH-treated spinal injured rats [n = 3 rats per experimental group analysed; 60–65 neurons analysed/time point/experimental condition at C6-7 (**A**); 80–85 neurons analysed/time point/experimental condition at L4-5 (**B**)]. Asterisks indicate significant differences between experimental groups; * p < 0.05.

We next investigated a target gene of HIF-1α, vascular endothelial growth factor (VEGF; [18]), a dimeric glycoprotein and fundamental regulator of angiogenesis that also appears to play neurotrophic and neuroprotective roles in spinal cord and brain injury [19–22]. AIH treatment + motor training for 7 days increased the expression of VEGF protein in spinal neurons at C6-7 and L4-5 segments of spinal cord, with no marked change in VEGF immunoreactivity following 1 day of treatment + training (Fig 4). Photomicrographs of spinal ventral grey matter processed for VEGF protein immunofluorescence show increased neuronal VEGF expression in AIH-treated spinal-injured rats relative to normoxia-treated spinal-injured rats after Day-7 of treatment in C6-7 (Fig 4A) and L4-5 (Fig 4B). Quantitative analysis confirmed that AIH treatment plus motor training for 7 days significantly increased protein expression of VEGF levels in neurons of ventral grey matter of spinal segments C6-7 and L4-5 in AIH-treated rats versus normoxia-treated rats (p < 0.05) (Fig 4A–4B and S2 Table).

### AIH treatment and motor training for 7 days resulted in higher neuronal expression of BDNF in cervical and lumbar neurons

**Fig 5:**
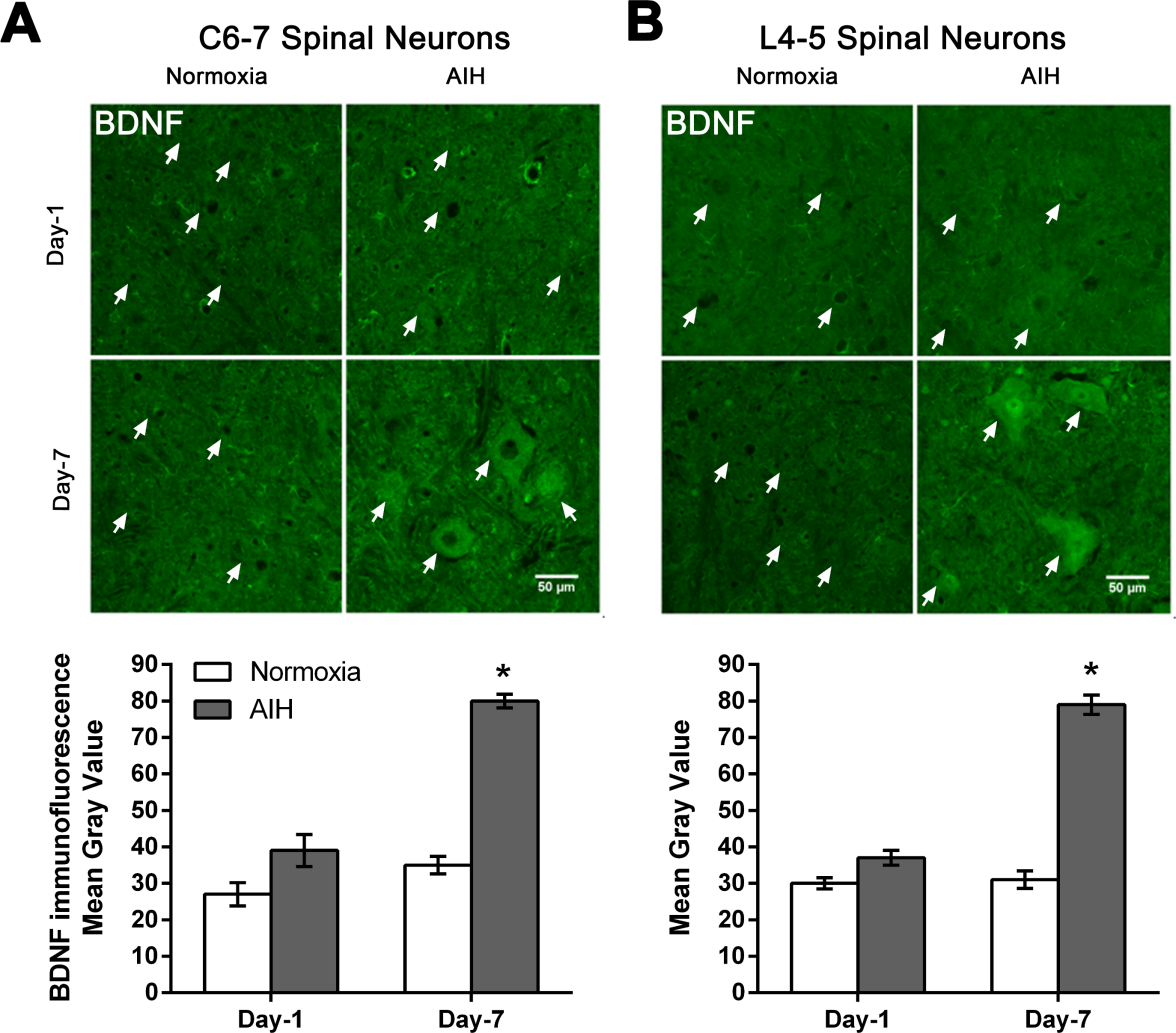
AIH treatment for 7 days increased BDNF protein levels in multiple spinal segments. Representative photomicrographs of the ventral grey matter in C6-7 (**A**) or L4-5 (**B**) spinal segments sections processed for BDNF immunofluorescence from 1 day (Day-1) or 7 day (Day-7) in normoxia-treated versus AIH-treated spinal injured rats. Arrows indicate representative neurons. Scale bar = 50 μm. Histograms summarize the mean immunofluorescence signal intensity detected ± SEM as measured in gray values over individual neurons within the ventral horn from normoxia- and AIH-treated spinal injured rats [n = 3 rats per experimental group analysed; 60–65 neurons analysed/time point/experimental condition at C6-7 (**A**); 80–85 neurons analysed/time point/experimental condition at L4-5 (**B**)]. Asterisks indicate significant differences between experimental groups; * p < 0.05.

BDNF was also examined due to its diverse role in modulating neural plasticity and enhancing functional recovery following SCI [23–29]. AIH treatment and motor training for 7 days increased the expression of BDNF protein in spinal neurons in both C6-7 and L4-5 segments of spinal cord relative to the normoxia controls and as detected using immunofluorescence (Fig 5). However, there was no discernible change in BDNF immunofluorescence over neurons at either cervical or lumbar spinal cord levels examined following 1 day of treatment and training (Fig 5). Quantitative analysis confirmed that the increase in BDNF immunofluorescence signal observed in neurons of C6-7 and L4-5 spinal segments in SCI rats in response to 7 day AIH and motor training was significantly higher than those detected in the normoxia plus motor training-treated SCI rats (p < 0.05) (Fig 5A–5B and S3 Table).

### AIH treatment and motor training for 7 days resulted in increased expression and activation of TrkB protein expression in ventral grey matter

**Fig 6:**
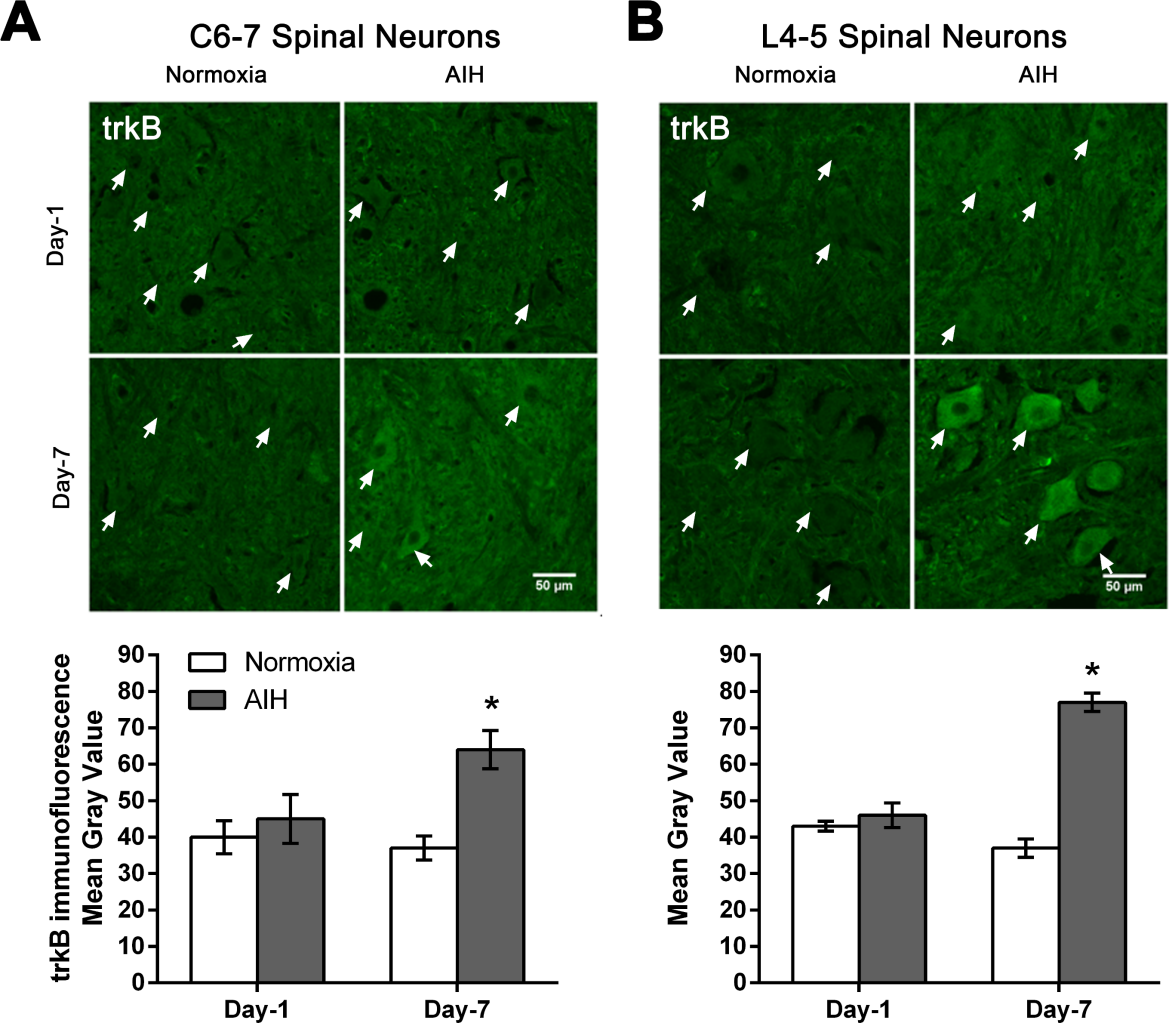
AIH treatment for 7 days increased TrkB protein levels in multiple spinal segments. Representative photomicrographs of the ventral grey matter in C6-7 (**A**) or L4-5 (**B**) spinal segments sections processed for TrkB immunofluorescence from 1 day (Day-1) or 7 day (Day-7) in normoxia-treated versus AIH-treated spinal injured rats. Arrows indicate representative neurons. Scale bar = 50 μm. Histograms summarize the mean immunofluorescence signal intensity detected ± SEM as measured in gray values over individual neurons within the ventral horn from normoxia- and AIH-treated spinal injured rats [n = 3 rats per experimental group analysed; 60–65 neurons analysed/time point/experimental condition at C6-7 (**A**); 80–85 neurons analysed/time point/experimental condition at L4-5 (**B**)]. Asterisks indicate significant differences between experimental groups; * p < 0.05.

BDNF signaling through its cognate receptor, tropomyosin-related kinase B (TrkB) [30] is a key pathway involved in activity-dependent processes driving neural plasticity. Thus, we next examined whether the elevations in neuron BDNF expression induced by AIH and motor training were also associated with increased expression of its receptor TrkB in this population of neurons. Immunofluorescence analysis of tissue sections at C6-7 and L4-5 spinal segments revealed that AIH treatment and motor training for 7 days effects increased the expression of TrkB protein in spinal neurons, with little change in expression noted after only one day of treatment (Fig 6). Quantitative analysis confirmed that these changes in TrkB expression in response to AIH treatment plus motor training for 7 days were significant (Fig 6 and S4 Table).

BDNF induces rapid phosphorylation of TrkB receptors [31–33], which is necessary for activation of many downstream actions. To assess whether the increased BDNF and TrkB expression effected by AIH and motor training was also associated with increased BDNF signaling in cervical and lumbar neurons, we processed additional tissue sections for immunofluorescence to detect alterations in the levels of tyrosine phosphorylated/activated TrkB.

**Fig 7:**
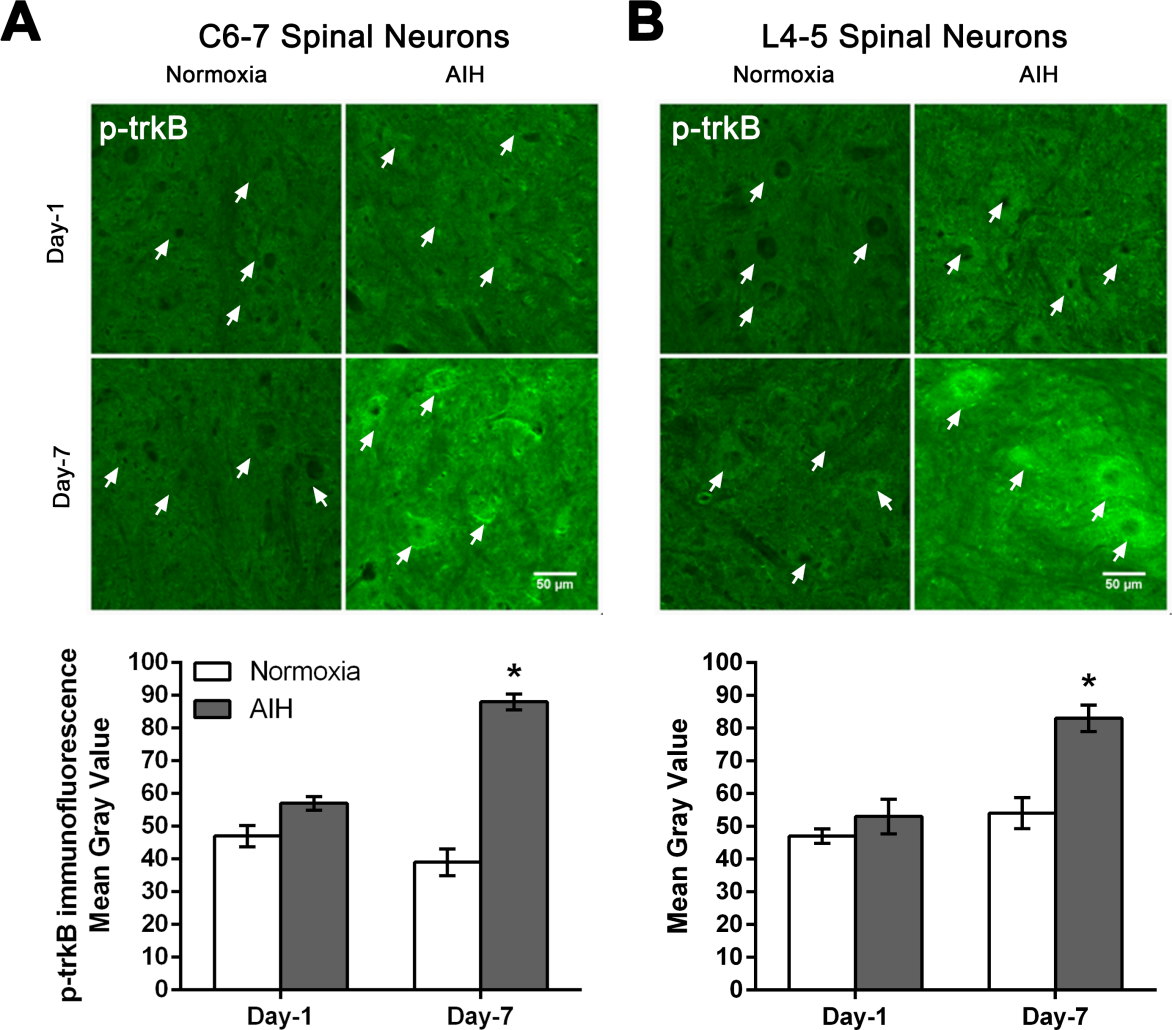
AIH treatment for 7 days increases phospho-TrkB (p-TrkB) protein levels in multiple spinal segments. Representative photomicrographs of the ventral grey matter in C6-7 (**A**) or L4-5 (**B**) spinal segments sections processed for p-TrkB immunofluorescence from 1 day (Day-1) or 7 day (Day-7) in normoxia-treated versus AIH-treated spinal injured rats. Arrows indicate representative neurons. Scale bar = 50 μm. Histograms summarize the mean immunofluorescence signal intensity detected ± SEM as measured in gray values over individual neurons within the ventral horn from normoxia- and AIH-treated spinal injured rats [n = 3 rats per experimental group analysed; 60–65 neurons analysed/time point/experimental condition at C6-7 (**A**); 80–85 neurons analysed/time point/experimental condition at L4–5 (**B**) ]. Asterisks indicate significant differences between experimental groups; * p < 0.05.

AIH treatment and motor training for 7 days in the SCI rats resulted in an increased phosphorylation and activation of TrkB protein in spinal neurons at C6-7 and L4-5 segments of spinal cord relative to normoxia and motor training treated control SCI rats (Fig 7). This increased phospho-TrkB immunofluorescence was already evident, albeit at low levels following one day of AIH treatment, with greatly increased levels observed after 7 days of AIH treatment at C6-7 (Fig 7A) and L4-5 (Fig 7B) spinal segment levels. The punctate pattern of staining observed at the level of the cell membrane and in the neuronal cytosol is consistent with activation at the membrane level, followed by internalization of the signaling endosome. Quantitative analysis confirmed that AIH treatment plus motor training resulted in significantly increased levels of phospho-TrkB detected in the C6-7 and L4-5 neurons from 7 day treated animals relative to normoxia-treated rats, but failed to reach significance in the one day AIH treated animals (Fig 7 and S5 Table).

## Discussion

In the present study, we assessed the combined effect of AIH and rehabilitative training on expression of hypoxia-associated (HIF-1α, VEGF) and plasticity-associated proteins (BDNF, TrkB, phospho-TrkB) in the spinal neurons of cervical SCI rats. AIH plus training administered for 7 days starting 4 weeks post-SCI significantly increased the expression of HIF-1α, VEGF, BDNF and TrkB molecules and enhanced TrkB activation in spinal neurons of cervical SCI rats at spinal segments C6-7 and L4-5. Unlike other proteins examined, the expression of the oxygen-sensitive transcription factor HIF-1α was also increased in spinal neurons after only 1 day of AIH treatment and motor training in SCI rats.

### AIH and motor training increased hypoxia-associated protein expression

The HIF-1 protein is a heterodimer, composed of two subunits: the constitutively expressed HIF-1β and HIF-1α expressed under hypoxia conditions [19, 34–36]. HIF-1 regulates the transcription of several dozen hypoxia-inducible target genes including VEGF, erythropoietin, inducible nitric oxide synthase, heme oxygenase-1, glucose transporter-1 and the glycolytic enzymes [35, 37–44]. Hypoxia induces the expression of HIF-1α in numerous cell types of the CNS including neurons, astrocytes, oligodendrocytes and microglia [45, 46].

The current finding that HIF-1α expression increased in spinal neurons after only one session of AIH treatment is consistent with the findings that exposure to low oxygen for only minutes to hours is sufficient to enhance HIF-1α levels. One hour of systemic hypoxia (6% O_2_) is sufficient to increase HIF-1α protein expression in brain tissues of mice, especially in the neurons of cerebral cortex and the granular layer of dentate gyrus in the hippocampus [47]. Only a few minutes of hypoxia induces the expression of HIF-1α proteins in the human epithelial carcinoma cell line HeLaS3, and after 1 hour of anoxia, HIF-1α protein expression reached its maximum level, and this maximum level was maintained for 4 hours [48]. Moreover, exposure to low oxygen (6% to 7% O_2_) for 30 minutes increased the expression of HIF-1α protein, but not its mRNA, 7 fold in rat retina, followed by a further increase after 3 hours of hypoxia to 15 fold compared to control animals [49]. Mice exposed to AIH for 120 minutes, comparable to the present study, showed increased HIF-1α expression in dorsal root ganglion neurons[14].

HIF-1α regulates the expression of VEGF, a 45kDa dimeric glycoprotein which is a fundamental regulator of pathological and physiological angiogenesis [19] but also plays neurotrophic and neuroprotective roles in the central and peripheral nervous systems [19, 50–54]. VEGF and its high affinity receptor VEGFR-2 are expressed in spinal neurons [55]. Repetitive AIH exposure (3×/week AIH: 10, 5-min episodes of 10.5% O_2_; 5-min normoxic intervals) upregulated the expression of VEGF and VEGFR-2 in respiratory and non-respiratory neurons in the spinal cord [13, 55, 56]. Consistent with these previous findings, the current study shows that AIH treatment for 7 days also significantly enhanced the expression of HIF-1α and VEGF at both C6-7 and L4-5 levels in the spinal cord (Figs 3 and 4). While AIH exposure for 1 day increased expression of HIF-1α, it was not sufficient to discernibly alter the expression of VEGF in spinal neurons. It is possible that a more sustained elevation in HIF-1α levels beyond that produced by 1 day of AIH treatment is required to induce downstream increases in VEGF expression.

Whether increased VEGF expression is related to improvements in ladder task performance seen in SCI rats after AIH + motor training is not yet known. In mice, 2 hours of *in vivo* AIH exposure results a HIF-1α -dependent improvement in axonal regeneration, along with hypoxia-induced JNK phosphorylation and upregulation of VEGF in dorsal root ganglion neurons[14]. In respiratory systems, exogenous application of VEGF *in vivo* elicits phrenic motor plasticity via the ERK/Akt intracellular pathways [1, 55, 56]. Collectively, these findings suggest that VEGF signalling might contribute to improved function in AIH-treated animals with nervous system injury [8, 9].

### AIH and motor training enhance the spinal expression of BDNF

Growth factors and trophic factors, including BDNF, play important roles in multiple forms of neural plasticity. BDNF mediates these effects through its high affinity receptor TrkB [57, 58]. Exogenous application of BDNF promotes neuroprotection, axonal regeneration, survival of neurons and axonal sprouting at the site of injury in SCI animals and has been reported to improve functional recovery after SCI [57–62]. Nevertheless, detrimental functional effects of exogenous BDNF have also been reported, including increased neuropathic pain and spasticity in SCI rats [63, 64]. Additionally, exogenous administration of BDNF must overcome difficulties related to access across the blood brain barrier, down-regulation of BDNF receptors, and triggering of immune responses [65, 66].

Increases in endogenous levels of BDNF might be more effective for facilitating functional recovery. Motor training following SCI enhances BDNF protein expression and this increase in BDNF expression is associated with locomotor functional recovery following SCI in animals [67–70]. Locomotor training, including voluntary wheel running and forced treadmill training, enhances the expression of BDNF protein and mRNA in spinal neurons and in skeletal muscles in spinal-injured rats [67, 71–76]. Furthermore, weight-supported treadmill training, either 100 or 1000 steps/training session daily for 4 weeks, has been shown to increase the BDNF expression in ventral grey matter of SCI rats [67]. Of particular interest to the current study, 4 weeks of treadmill training initiated 1 month after injury increased BDNF expression in the lumbar spinal neurons caudal to a complete spinal transection in rats [73]. In the current study, 1 week of ladder training initiated 4 weeks after spinal injury was not sufficient to increase BDNF expression in cervical or lumbar neurons (Fig 5, Day 7-Normoxia). Instead, 1 week of AIH administered in addition to motor training increased BDNF levels in spinal neurons (Fig 5, Day 7-AIH).

Consistent with our findings, several different protocols of AIH (daily for 7 days or 3×/week for 10 weeks) have shown increased expression of BDNF and TrkB as well as enhanced activation of TrkB (phospho-TrkB) in spinal motor nuclei [8, 13, 56, 77]. Importantly, brief episodes of hypoxia in intact rats initiated the synthesis of new BDNF in the cervical cord, and this BDNF was sufficient and necessary to induce respiratory plasticity [77, 78]. The current study extends these previous findings by confirming that BDNF expression also increases at multiple spinal sites after 7 days of treatment with AIH treatment plus motor training.

### AIH and motor training enhance the spinal expression of TrkB and its activation

The BDNF receptor TrkB is a transmembrane proteins of the Trk family of neurotrophin receptors [30]. TrkB receptors are activated by BDNF-induced formation of receptor dimers, whereupon the dimerized receptors rapidly phosphorylate each other and activate downstream signaling pathways [79–81]. In the current study, the increased spinal expression of TrkB and its subsequent phosphorylation/activation after 7 days of AIH and motor training in SCI rats strongly suggests that the effects of this combined treatment are at least in part mediated by the activation of TrkB signalling pathways. This is consistent with increased BDNF expression described above, as well as with previous findings of increased TrkB expression and its activation after repetitive AIH treatment in intact rats, and AIH-induced activation of TrkB receptors in spinal neurons resulting in increased output of the phrenic nerve [8, 56, 77, 78, 82].

## Conclusion

In the present study, we have shown that a low-dose protocol of AIH treatment + motor training in SCI rats produced temporal and spatial differential expression of hypoxia- and plasticity-related proteins in spinal neurons. The pattern of expression of these proteins occurs under identical experimental conditions which result in recovery of skilled forelimb use in SCI animals. Together with the capacity of AIH and motor training to improve walking abilities in persons with chronic incomplete SCI, these findings suggest that AIH has potential as an effective therapy to restore motor function after nervous system injury.

## Supporting information

S1 Table ImageJ-quantified gray scale values of immunostained spinal neurons using anti-anti-HIF-1α antibody. (XLSX)

S2 Table ImageJ-quantified gray scale values of immunostained spinal neurons using anti-VEGF antibody. (XLSX)

S3 Table ImageJ-quantified gray scale values of immunostained spinal neurons using anti-BDNF antibody. (XLS)

S4 Table ImageJ-quantified gray scale values of immunostained spinal neurons using anti-TrkB antibody. (XLSX)

S5 Table ImageJ-quantified gray scale values of immunostained spinal neurons using anti-phopho-TrkB antibody. (XLSX)
